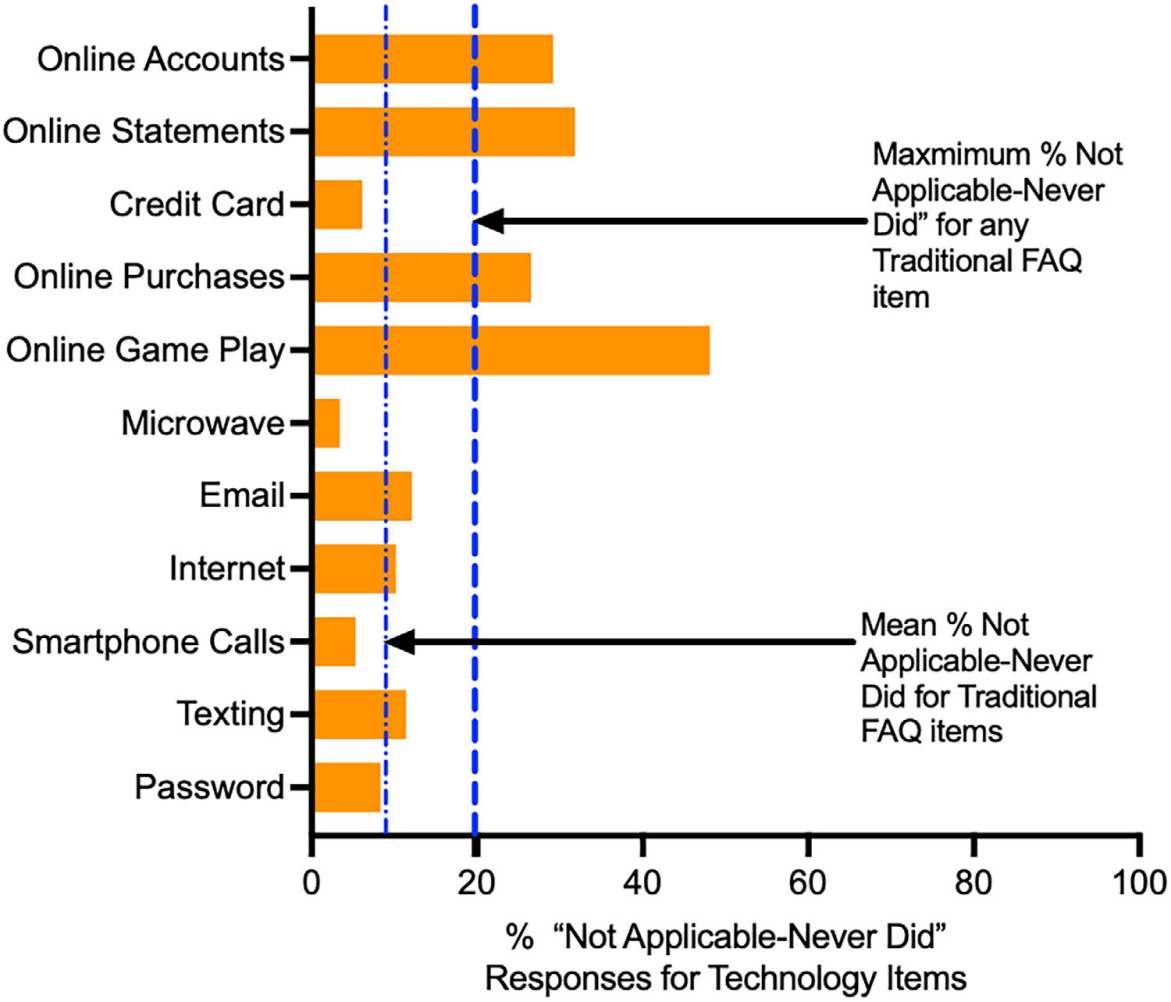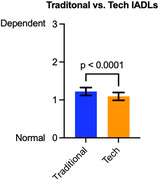# Measuring technology use as an instrumental activity of daily living in those with late life cognitive disorders

**DOI:** 10.1002/alz.086115

**Published:** 2025-01-03

**Authors:** Jared F Benge

**Affiliations:** ^1^ University of Texas–Austin, Austin, TX USA

## Abstract

**Background:**

Older adults increasingly rely on digital technologies to perform instrumental activities of daily living (iADLs), including commerce, managing accounts online, using texting and websites for social connection, and accessing health services via web platforms. Despite the increasingly central role of technology to daily life, current iADL measures do not regularly capture the digital approach to daily activities. The current study had three broad aims 1) determine the applicability of technology‐based iADLs to the daily lives of older adults being evaluated for Alzheimer’s disease and related dementias (ADRD), 2) compare the level of dependence for tech and traditional iADL items, and 3) determine if adding technology related iADL items improves the diagnostic accuracy of iADL assessments.

**Methods:**

264 care partners (58.7% spouses) of older adults referred for evaluations of cognitive disorders (34.5% with MCI; 51.9% with dementia) completed the Functional Activities Questionnaire (traditional ADL measure) and 11 technology‐based iADL items (i.e. remembering passwords, managing online accounts, using a device for messaging, etc.).

**Results:**

Rates of “not applicable, never did” were uncommon for technology‐based items, including microwave use (3.8%), smartphone use for calls (5.3%), and remembering passwords (8.3%). Increasing age and decreasing education predicted more not applicable responses to technology items. Average dependence ratings of technology items were less than for traditional ADLs (*t(*263) = 4.29, *p*<.0001; figure 2), and increased as a function of ADRD severity. Adding technology ‐based items marginally increased diagnostic accuracy of ADL measures for detecting dementia (AUC .85 vs. .83; *z* = ‐2.07, *p* = .03).

**Conclusions:**

Technology use amongst older adults presenting for evaluations of cognitive decline is common for a range of daily activities. That being said, a digital divide still exists, with older age and less education predicting less applicability of technology‐based iADLs. Despite stereotypes to the contrary, overall degree of dependence for technology was less than for traditional, largely analog based iADLs, likely due to the ability to automate some tasks (i.e., automatic bill pay). We discuss how these findings broadly fit in the larger technological reserve hypothesis, implications for clinical assessment of iADLs, and approaches for future proofing iADL measures.